# Structure, function and five basic needs of the global health research system

**DOI:** 10.7189/jogh.06.010505

**Published:** 2016-06

**Authors:** Igor Rudan, Devi Sridhar

**Affiliations:** Centre for Global Health Research and WHO Collaborating Centre for Population Health Research and Training, The Usher Institute for Population Health Sciences and Informatics, University of Edinburgh, Scotland, UK

## Abstract

**Background:**

Two major initiatives that were set up to support and co–ordinate global health research efforts have been largely discontinued in recent years: the Global Forum for Health Research and World Health Organization's Department for Research Policy and Cooperation. These developments provide an interesting case study into the factors that contribute to the sustainability of initiatives to support and co–ordinate global health research in the 21st century.

**Methods:**

We reviewed the history of attempts to govern, support or co–ordinate research in global health. Moreover, we studied the changes and shifts in funding flows attributed to global health research. This allowed us to map the structure of the global health research system, as it has evolved under the increased funding contributions of the past decade. Bearing in mind its structure, core functions and dynamic nature, we proposed a framework on how to effectively support the system to increase its efficiency.

**Results:**

Based on our framework, which charted the structure and function of the global health research system and exposed places and roles for many stakeholders within the system, five basic needs emerged: (i) to co–ordinate funding among donors more effectively; (ii) to prioritize among many research ideas; (iii) to quickly recognize results of successful research; (iv) to ensure broad and rapid dissemination of results and their accessibility; and (v) to evaluate return on investments in health research.

**Conclusion:**

The global health research system has evolved rapidly and spontaneously. It has not been optimally efficient, but it is possible to identify solutions that could improve this. There are already examples of effective responses for the need of prioritization of research questions (eg, the CHNRI method), quick recognition of important research (eg, systems used by editors of the leading journals) and rapid and broadly accessible publication of the new knowledge (eg, *PLoS One* journal as an example). It is still necessary to develop tools that could assist donors to co–ordinate funding and ensure more equity between areas in the provided support, and to evaluate the value for money invested in health research.

In the past four years, two major initiatives that were set up with the aim to support and co–ordinate global health research efforts have been largely discontinued. The first is the Global Forum for Health Research, which was established in Geneva in 1998 to support WHO’s focus on health research [1]. The second is WHO's Department for Research Policy and Cooperation (WHO RPC), which ceased its operations in 2012 during the WHO's internal reform. Almost ironically, the annual WHO World Health Report for 2012 announced its theme as: “*No health without research*” and was to be coordinated by the WHO RPC [2]. The journal *PLoS Medicine* agreed to publish a special series on health research in parallel to the release of the World Health Report, as discussed in the journal's editorial to the series, entitled: “*The World Health Report 2012 that Wasn’t*” [3]. Eventually, the report was retitled “*Research for Universal Health Coverage*” and published in 2013 [4].

These developments provide an interesting case study into the factors that contribute to the sustainability of initiatives to govern, support and co–ordinate global health research in the 21st century. A timeline of key events that set the current context is shown **Figure 1**. In this viewpoint, we will map the structure of the global health research system as it has evolved under the funding increases of the past decade. Bearing in mind its structure, core functions and dynamic nature, we will propose a framework on how to effectively support the system to increase its efficiency.

**Figure 1:**
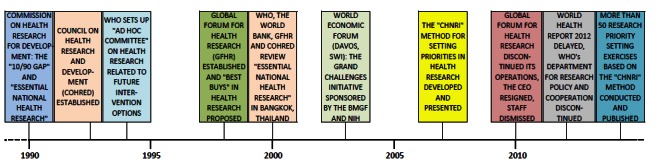
A timeline of several important events relevant to governance, support and co–ordination of global health research that determined the current context.

## THE EVOLVING STRUCTURE OF THE GLOBAL HEALTH RESEARCH SYSTEM

Over the past two decades, the funding available for health research has increased rather dramatically from US$ 50 billion in 1993 to US$ 240 billion in 2009 [5], but this did not happen in any planned or coordinated way. Those who tried tracking this funding – such as the Global Forum for Health Research in its annual reports, G–FINDER, the Institute for Health Metrics and Evaluation and other academics, provided rather different figures [5–9]. This discrepancy is largely due to the difficulty in distinguishing research funding from broader development assistance for health. There is also lack of consensus on whether the funding invested in high–income countries to study health challenges that may be relevant to low and middle–income countries should also be included. Still, under any assumption, the interest in funding global health research is growing, and the structure of this system is rapidly evolving.

**Figure 2:**
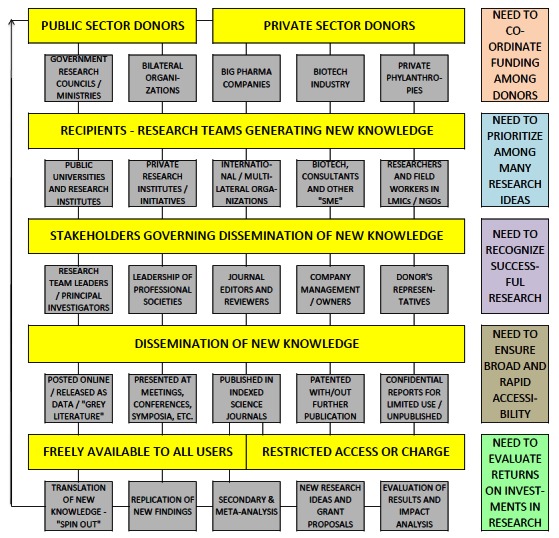
The structure of the global health research system and the five basic needs to ensure its efficient performance.

In **Figure 2**, we show the simplified representation of the key stakeholders and processes, based on how the funds flow through the system. At the beginning of the system is the source of the funding – with donors being either public, private, or the emerging “class” of donors – the large philanthropies, such as the Bill and Melinda Gates Foundation (BMGF), the Carlos Slim Foundation, and the Rockefeller Foundation. They all provide financial support for the projects of researchers employed in universities, research institutes, international organizations, biotech companies and small and medium enterprises (SME are a growing “class” of recipients). They also fund stakeholders with research capacity in low and middle–income countries that can help carry out the research projects as equal partners. Eventually, the responsibility for spending the funds is passed down to research teams and their international consortia, which conduct research to generate new knowledge in several generic areas: measuring a problem; understanding its cause(s); elaborating solutions; translating the solutions or evidence into policy, practice and products; and/or evaluating the effectiveness of solutions [10].

The decision over the channel of dissemination of this knowledge is made by a new set of stakeholders (**Figure 2**), which may involve research committees of public institutions, journal editors, reviewers, donor representatives, company managers or owners. The bulk of work will end up published by research journals, where editors and reviewers, and sometimes even private publishers, influence decisions on the shape and form of publication. The funders increasingly require researchers to publish in open–access journals. Some of the findings do not get published because placing the knowledge in the public domain would invalidate patent applications and subsequent financial profits. This new knowledge can also be presented at conferences, published as a report to the funder, as “grey literature”, or simply posted on the internet. Finally, in many cases, new knowledge does not get published in any way – perhaps due to insufficient relevance or novelty, concerns over its quality, or simply a lack of a positive result. In the end, the published knowledge can be professionally evaluated and replicated, with a growing industry of companies offering those services. Moreover, universities have set up structures to help researchers to commercialize their work and set up spin–out companies.

## THE CORE FUNCTIONS OF THE GLOBAL HEALTH RESEARCH SYSTEM

There should not be much controversy over the main function of the global health research system: it is there to use donors’ funding to support experiments that address pertinent health research questions. In this way, through answering those questions, new knowledge is continuously being generated. This knowledge is then translated into both clinical and public health practice in order to reduce the burden of disease in the population and improve health–related outcomes.

The effectiveness of the global health research system to perform its main function will depend on the efficiency of several of its sub–components (**Figure 2**). First, donors need to be *motivated* to continue investing; *informed* to understand the targets; and *coordinated* to avoid over– and under–funding certain areas. This, in turn, ensures efficiency of their investments. Second, researchers need to prioritize research ideas well, to balance those that could benefit the public relatively soon with more speculative and downstream ones. They need to design and conduct the experiments carefully to ensure that their efforts are useful even when the result is negative. Third, managers, journal editors and media need to recognize important progress accurately to ensure efficiency in selection of work that receives attention. Fourth, publishers need to ensure broad open access to all new knowledge that results from health research and rapid accessibility of information without exception. Fifth, the effectiveness of translation of the new knowledge into practice needs to be evaluated. This is important because it could help recognizing the most promising research projects and ideas earlier in the process. It would also allow comparisons of returns on investments in health research with other competing investments that could also improve health, such as development assistance, infrastructure projects, or simply increased purchase and coverage of existing interventions.

After the relatively stagnant nature of the global health research system throughout most of the second half of the 20th century, the system evolved rapidly over the past decade and took a life of its own in all of its segments. Attempts to support and co–ordinate such a dynamic and unpredictably evolving system using a ‘top–down’ approach may have seemed a feasible and sustainable mission from the perspective of the post–World War II world, when the UN was established. However, the 21st century global health research system has developed in a “bottom–up”, “laissez–faire” manner, in which the stakeholders themselves are continuously inventing improved practices and introducing changes in the models that worked well in previous decades. This is happening at all levels – with emerging big donors, innovative finance mechanisms, creative organization of large international consortia of research teams and their collaborations on “big science”. There are now many web–based routes to publication, new tools and measures of assessment of research output (like *Google Scholar, Scopus, Research Gate* and *H–index* metric), and increased support mechanisms for rapid translation, commercialization and implementation of research results. In such a dynamic system, any attempt to influence the relevant stakeholders and processes from the “outside” by a group of experts who drive their legitimacy exclusively from a fact that they are employees or affiliates of the UN is largely unrealistic and outdated.

## FIVE BASIC NEEDS OF THE GLOBAL HEALTH RESEARCH SYSTEM AND PROPOSED SOLUTIONS TO IMPROVE ITS EFFICIENCY

We now propose an alternative route to improved efficiency of the global health research system that would be primarily needs–based, and therefore likely welcomed by the stakeholders in the system. At the top of **Figure 2**, it is clear that the emergence of new donors is certainly a positive development, but it requires their sustained motivation and also carries a large risk of becoming un–coordinated and unbalanced, with high preference towards certain topics and neglect of others. This is a real risk that has already been exposed in even the most basic analysis of funding flows [7]. To help the system develop and grow in an equitable way at this level, there is a need to continuously track funding using an internationally agreed methodology, preferably by more than one agency/institute. Beyond simply tracking funding, a tool is needed to ensure that no areas are neglected in comparison to areas of strong donor preference, thus assisting policy–makers and donor representatives. As a possible solution, we are working to propose a “Stock Market for Global Health Research Investment Options” – a tool that would use analogy to real–time stock markets to compare the burden of different health problems with the investments being committed to those problems, using the most recent available information.

The main need at the level of the recipients in the system – the communities of researchers (**Figure 2**) – is to find ways to communicate and agree on their own field's research priorities, so that a more balanced and unified case on funding priorities could be presented to donors from the “cutting edge” of research. As a possible solution, “*the CHNRI method”* developed by the Child Health and Nutrition Research Initiative (CHNRI) of the Global Forum for Health Research seems to be an example of this need being met rather effectively [11]. This “crowd–sourcing” approach to generating and managing research ideas, while balancing short–term and long–term vision and different instruments of health research, has been validated through many applications [12–15]. The results from 50 conducted research prioritization exercises have been published by mid–2015, and many further exercises are being conducted presently [15]. A recent independent review showed that 18% of prioritization exercises in global health research in recent years used the CHNRI methodology, which made it the most frequently used specific priority–setting method [16].

Then, at the level of stakeholders who govern dissemination of research results (**Figure 2**), there is a need for a tool, process or a system that would recognize important research, promote and reward it appropriately [17]. Interestingly, journal editors operate such systems already while reaching their decisions on which papers to publish. Given that many of them select less than 10% of submissions for publication, the journals that manage to maintain high quality and substantial impact over time have clearly developed well–performing systems. We propose to learn more of their decision–making systems and processes and review the results of their work – both at the level of journal's impact, and of individual papers – over long periods of time. This should allow development of a system that would be highly sensitive to important research and ensure its publication, but also quite specific, reducing the amount of published work that is not relevant.

Clearly, it is difficult to predict the impact that research articles may have in the future at the point at which they are being evaluated. However, in the new world of “big data”, it is possible to conduct massive exercises in available databases of research papers and their received citations to search for common patterns that are shared among those papers that have most impact. Recently, the journal *Nature* devoted a special news feature to analysis of the 100 most cited papers of all time [18]. In a related feature, titled “Is your most cited work your best?”, Ioannidis et al. tried to capture the key dimensions that need to be addressed to make any biomedical research “exceptional”. They asked about 400 most cited biomedical scientists in the world (123 of whom responded) to score their 10 most cited papers from 0–100 for each of the six criteria that they hypothesized may be inherent to truly exceptional work. They termed these six criteria “Continuous Progress, Broader Interest, Greater Synthesis, Disruptive Innovativeness, Surprise and Publication Difficulty” [19]. Their exercise made some of the first steps towards a more systematic and transparent framework that could allow capturing the exceptional nature of biomedical research articles at the time they are evaluated, rather than having to wait for many years to determine their importance through impact they generated and citations they received [19].

At the next level – dissemination of new knowledge (**Figure 2**) – the need for broad and rapid access to new knowledge is presently being addressed through the “open access” movement, world wide web development, IT–based solutions for publication, dissemination and search engines, social networks and internet–based media [20]. The success of *PLoS One* journal can be used as an excellent example. We believe that the journal succeeded in a very short time, and well beyond expectations, precisely because it provided an effective solution to this particular need of the global health research system. It is enough to state that in the year of its inception, in 2006, it published 137 papers; in 2007 it already published 1230 papers, and in 2013 a staggering 31 498 papers, with the number per year still growing strongly. At the same time, given an unprecedentedly large denominator, it still manages to keep a very decent impact factor of around 4.0 in the past several years. Clearly, many participants in the global health research system have recognized *PLoS One* as a solution that addresses one of the system's major needs.

Finally, at the level of research outputs, a tool is needed that could evaluate returns on investments in global health research, and what is seen as the value for money gained through those investments [21]. The tool should also monitor success rates in translation and implementation of the outcomes into products and programmes, all the way to measurable benefits for global public health. Such a tool would allow a proper understanding of the actual value of investing in health research, in comparison to alternative forms of investments that can also benefit health – eg, community infrastructure projects, improved education, safety, social welfare, and transportation. It is perhaps time to get some understanding on whether the many trillions invested in health research have been a reasonable investment – especially in the wake of Big Pharma largely closing down their R&D departments, which may provide an indication that they are concerned about the feasibility of those investments in comparison to alternatives. This need will be the most difficult to address, but we aim to propose a draft solution and keep improving it over time.

## CONCLUSION

The global health research system has evolved rapidly and spontaneously. It has not been optimally efficient, but it is possible to identify solutions that could improve this. There are already examples of effective responses for the need of prioritization of research questions (eg, the CHNRI method), rapid recognition of important research (eg, systems used by editors of the leading journals) and quick and broadly accessible publication of the new knowledge (eg, *PLoS One* journal as an example). It is still necessary to develop tools that could assist donors to co–ordinate funding and ensure more equity between areas in the provided support, and to evaluate the value for money invested in health research.
